# The association between serum glucose to potassium ratio on admission and short-term mortality in ischemic stroke patients

**DOI:** 10.1038/s41598-022-12393-0

**Published:** 2022-05-17

**Authors:** Yuzhao Lu, Xin Ma, Xiaobing Zhou, Yang Wang

**Affiliations:** 1 Department of Neurosurgery, The First Affiliated Hospital of Nanchang University, Nanchang University, Yongwai Zheng Road 17, Nanchang, 330006 China; 2 Department of Thoracic Surgery, Jingshan Union Hospital of Huazhong University of Science and Technology, Jingshan, 431899 Hubei China; 3 Present Address: Department of Neurosurgery, Beijing Chaoyang Hospital, Capital Medical University, South Gongti Road 8, Chaoyang District, Beijing, 10043 China

**Keywords:** Diseases, Neurology, Risk factors

## Abstract

High serum glucose to potassium ratio (GPR) at admission is implicated for a poor outcome in acute brain injury, acute intracranial hemorrhage, and aneurysmal subarachnoid hemorrhage. However, the relationship between GPR and the outcome of ischemic stroke (IS) remains unknown. In all, 784 IS patients from a large emergency Norwegian cohort were included for secondary analysis. The exposure and outcome were GPR at baseline and all-cause mortality within 30 days after the first admission. Multivariable logistic regression analysis was performed to estimate the risk of 30-day mortality based on GPR levels. In addition, we examined whether there was a nonlinear relationship between admission GPR and 30-day mortality using two-piecewise linear regression with a smoothing function and threshold level analysis. The results of multivariable regression analysis showed that GPR at baseline was positively associated with the 30-day mortality (OR 2.01, 95% CI 1.12, 3.61) after adjusting for potential confounders (age, gender, department, serum sodium, serum albumin, serum-magnesium, hypertension, heart failure, chronic renal failure, and pneumonia). When GPR was translated to a categorical variable, the ORs and 95% CIs in the tertiles 2 to 3 *versus* the tertile 1 were 1.24 (0.60, 2.56) and 2.15 (1.09, 4.24), respectively (P for trend = 0.0188). Moreover, the results of the two-piecewise linear regression and curve fitting revealed a linear relationship between GPR and 30-day mortality. In IS patients, GPR is positively correlated with 30-day mortality, and the relationship between them is linear. The GPR at admission may be a promising predictor for the short-term outcome in IS patients.

## Introduction

Ischemic stroke (IS) is a commonly acute and severe disease with high mortality and disability rates, imposing an increasingly heavy socioeconomic burden globally^1^. Currently, intense efforts are being made to find novel risk predictors, which are simple and easily accessible, to better guide clinical decision-making for patients with IS. Circulating biomarkers in blood samples were clinically very common; thus, the relationship between circulating biomarkers and prognosis has gained increasing attention for stroke in recent years^2,3^.

Serum glucose and potassium are two important blood indicators that are commonly used clinically. As the main energy source of cells in the human body, glucose is a critical factor for maintaining cellular metabolism^4^. Potassium ion, the most abundant cation in the cells of a human body, plays a crucial role in physiological processes including neural conduction, cardiac pulsation, muscle contraction, and maintenance of normal renal function^5^. In addition, both serum glucose and potassium disturbances have been revealed to be correlated with the risk of stroke^6,7^. Previous studies have demonstrated that there were complex interactions among potassium and glucose in the human body^8,9^. Given the potential combined effects of glucose and serum potassium, the serum glucose to potassium ratio (GPR) has been used in a few studies and has been shown to be an early prognostic factors for central nerve injures including aneurysmal subarachnoid hemorrhage (aSAH)^10^, acute intracerebral hemorrhage^11^, severe traumatic brain injury^12^, and neuropsychiatric syndrome after carbon monoxide poisoning^13^.

However, the relationship between GPR and the clinical outcome of IS remains unknown. Therefore, we aimed to explore the association between GPR at admission and short-term mortality in IS patients based on a retrospective cohort study.

## Methods

### Data source

Original data were published by Tazmini et al.^14^ on the “DRYAD” website (www.datadryad.org). And Tazmini et al.^15^ authorized the ownership of their raw data to the “DRYAD” database. Thus, this secondary research based on the raw data for a different research hypothesis was permitted.

**Figure 1:**
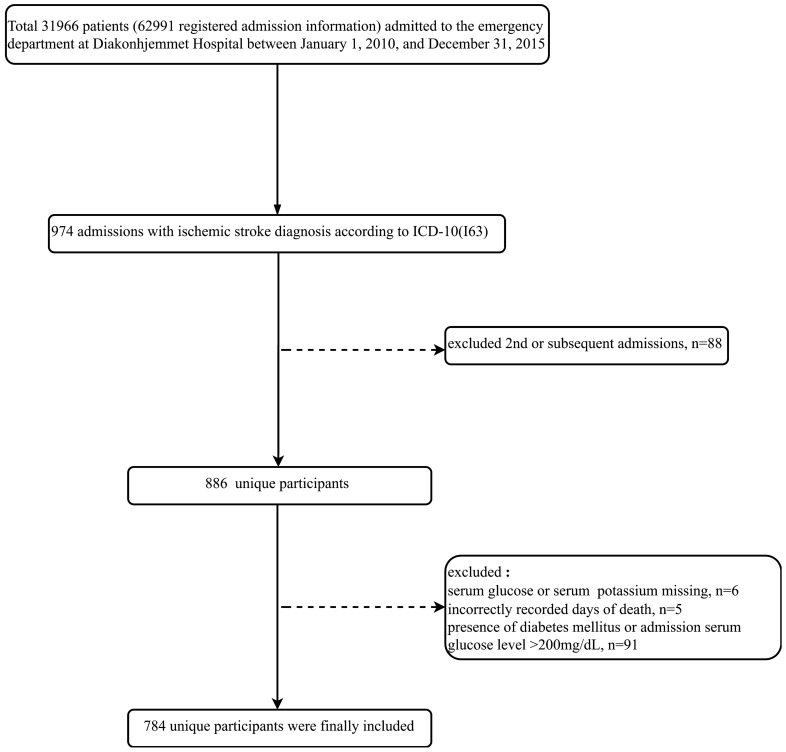
Flowchart of screening the study population.

The original research was a single-center retrospective cohort study that included 31,966 unique patients (62,991 registered admission information) who visited the emergency department of the Diakonhjemmet Hospital in Oslo (Norway) from 2010 to 2015. According to the ICD-10 standard of classification, 974 visits (admission information) were diagnosed as IS (ICD-10, I63). The raw data included information on multiple hospitalizations for the same patient, but only the first visit of each patient was considered in this study. Thus, we excluded the second or subsequent admissions (n = 88). Only 886 unique IS patients were then considered during analysis. Subsequently, 102 patients were excluded for missing data concerning serum glucose or potassium levels (n = 6), incorrectly recorded days of death (n = 5), and presence of diabetes mellitus or serum glucose level > 200 mg/dL (n = 91) at admission. Finally, 784 unique participants were included in the study (Fig. 1).

### Exposure

All the laboratory indicators were obtained from the first-time laboratory results at admission. Serum calcium (mmol/L), serum-albumin (g/L), serum-sodium (mmol/L), serum-potassium (mmol/L), serum-glucose (mmol/L), serum-phosphate (mmol/L), and serum-magnesium (mmol/L) were recorded in the original data. The serum glucose to potassium ratio was calculated as serum glucose concentration (mmol/L) divided by serum potassium concentration (mmol/L)^12^.

### Co-morbidities and other variables

Secondary diagnostic information was used to identify co-morbidities including diabetes (ICD-10: E10–E14), hypertension (ICD-10: I10), hyperlipemia (ICD-8: E78), atrial fibrillation/atrial flutter (ICD-10: I48), heart failure (ICD-10: I50), acute renal failure(ICD-10: N17), chronic renal failure (ICD-10: N18), chronic obstructive pulmonary disease (ICD-10: J42–44), coronary heart disease (ICD-10: I25), chronic obstructive pulmonary disease (ICD-10: J42–44), cancer (ICD-10: C0–C9, Z51.0-3), malnutrition (ICD-10: E40–E46), and pneumonia (ICD-10: J98, J69, J11–18).

All IS patients were categorized into five subtypes according to the TOAST stroke subtype classification system: large-artery atherosclerosis, cardioembolism, small-vessel occlusion, other determined etiology, and undetermined^16^. The information of patients admitted to the medical or surgical department was identified as a binary variable.

### Outcome

The primary outcome was all-cause mortality within 30 days after first admission.

### Missing data

All missing data of covariates are stated in Table 1. Considering that missing data may reduce statistical power or even lead to bias, covariates with too much missing data (e.g., concerning serum phosphate and serum magnesium levels) were handled as categorical variables. And dummy variables were used to identify the missing values of the covariate^17^.

**Table 1:** Baseline characteristics of subjects according to tertiles of GPR level.

GRP tertiles	T1 (≤ 1.372)	T2 (1.375–1.658)	T3 (≥ 1.659)	P-value
N = 784	258	263	263	
Age (years)	77.10 ± 12.98	77.35 ± 12.15	78.46 ± 11.81	0.518
Gender (male), n (%)	110 (42.64%)	118 (44.87%)	113 (42.97%)	0.857
**Ischemic stroke subtypes (TOAST)**				0.903
Large-artery atherosclerosis	20 (7.75%)	23 (8.75%)	28 (10.65%)	
Cardioembolism	21 (8.14%)	27 (10.27%)	22 (8.37%)	
Small-vessel occlusion	11 (4.26%)	9 (3.42%)	10 (3.80%)	
Other determined etiology	0 (0.00%)	1 (0.38%)	1 (0.38%)	
Undetermined	206 (79.84%)	203 (77.19%)	202 (76.81%)	
**Department**				0.369
Medical	257 (99.61%)	263 (100.00%)	261 (99.24%)	
Surgical	1 (0.39%)	0 (0.00%)	2 (0.76%)	
Serum-sodium (mmol/L)	139.84 ± 3.37	139.51 ± 3.23	139.17 ± 4.12	0.105
Serum-calcium (mmol/L) (1 missing)	2.34 ± 0.11	2.35 ± 0.13	2.35 ± 0.13	0.428
Seum-albumin (mmol/L) (2 missing)	39.44 ± 3.91	39.28 ± 3.67	38.92 ± 4.03	0.164
**Serum-phosphate tertiles (mmol/L), n (%)**				0.045
Tertile1 (≤ 0.97)	17 (6.59%)	38 (14.45%)	30 (11.41%)	
Tertile2 (0.98–1.12)	25 (9.69%)	26 (9.89%)	37 (14.07%)	
Tertile3 (≥ 1.13)	39 (15.12%)	32 (12.17%)	29 (11.03%)	
Not recorded	177 (68.60%)	167 (63.50%)	167 (63.50%)	
**Serum-magnesium tertiles (mmol/L), n (%)**				0.312
Tertile1 (≤ 0.78)	19 (7.36%)	30 (11.41%)	38 (14.45%)	
Tertile2 (0.79–0.84)	30 (11.63%)	32 (12.17%)	27 (10.27%)	
Tertile3 (≥ 0.85)	37 (14.34%)	34 (12.93%)	36 (13.69%)	
Not recorded	172 (66.67%)	167 (63.50%)	162 (61.60%)	
Hyperlipemia, n (%)	13 (5.04%)	10 (3.80%)	7 (2.66%)	0.368
Hypertension, n (%)	61 (23.64%)	66 (25.10%)	64 (24.33%)	0.928
AF, n (%)	60 (23.26%)	64 (24.33%)	85 (32.32%)	0.038
Heart failure, n (%)	7 (2.71%)	10 (3.80%)	6 (2.28%)	0.567
Chronic renal failure, n (%)	10 (3.88%)	11 (4.18%)	12 (4.56%)	0.926
Acute renal failure, n (%)	5 (1.94%)	6 (2.28%)	7 (2.66%)	0.859
COPD, n (%)	3 (1.16%)	5 (1.90%)	2 (0.76%)	0.497
CHD, n (%)	14 (5.43%)	18 (6.84%)	10 (3.80%)	0.301
Cancer, n (%)	0 (0.00%)	7 (2.66%)	7 (2.66%)	0.030
Malnutrition, n (%)	5 (1.94%)	7 (2.66%)	6 (2.28%)	0.859
Dehydration, n (%)	8 (3.10%)	6 (2.28%)	15 (5.70%)	0.095
Pneumonia, n (%)	6 (2.33%)	12 (4.56%)	18 (6.84%)	0.048
30-day mortality	18 (6.98%)	22 (8.37%)	36 (13.69%)	0.024

All data in GPR subgroups were expressed as mean ± SD or number (%). GPR tertiles: Tertile 1: ≤ 1.372; Tertile 2: 1.375–1.658; Tertile 3: ≥ 1.659. Serum-phosphate tertiles: Tertile 1: ≤ 0.97 mmol/L; Tertile 2: 0.98–1.12 mmol/L; Tertile 3: ≥ 1.13 mmol/. Serum-magnesium tertiles: Tertile 1: ≤ 0.78 mmol/L; Tertile 2: 0.79–0.84 mmol/L; Tertile3: ≥ 0.85 mmol/L.

*GPR* serum glucose to potassium ratio, *AF* atrial fibrillation/atrial flutter, *COPD* chronic obstructive pulmonary disease, *CHD* coronary heart disease.

In addition, to further assess whether missing data being handled as dummy variables can introduce bias into the results, we used multiple imputations based on five replications and chained equation approach in the R MI procedure to handle all the missing data for sensitivity analysis^18^. The process of multiple imputation and the results of multivariate regression analysis based on five multiple imputation data are shown in Supplementary Fig. 1 and Supplementary Table 1 respectively.

### Statistical analysis

All analyses were performed with EmpowerStats (www.empowerstats.com, X&Y Solutions, Inc., Boston, MA) and the statistical software package R (http://www.R-project.org, The R Foundation). P < 0.05 was considered statistically significant.

Continuous and categorical variables are expressed as Mean ± SDs and percentages, respectively. To examine the differences among subgroups of variables, we used one-way ANOVA test for continuous variables with normal distribution, Kruskal–Wallis H test for continuous variables with skewed distribution, and chi-square test (or Fisher’s exact test) for categorical variables.

Multiple logistic regression analysis was used to explore the association between GPR and outcome (30-day mortality); OR and 95% CI were used for risk evaluation. To evaluate whether there is a potential non-linear relationship between GPR and 30-day mortality, curve fitting and two-piecewise linear regression analysis were performed.

We built three models to regulate the potential confounding factors, they are : (1) Crude model, i.e., unadjusted; (2) Model I, adjusted for age and gender; (3) Model II, adjusted for age, gender, department, serum sodium, serum albumin, serum-magnesium tertiles, hypertension, heart failure, chronic renal failure, and pneumonia. Covariates were selected based on their relationship to 30-day mortality or their ability to change the effect value by more than 10%^19^, gender was also included as a basic covariate.

### Sensitivity analysis

GPR tertiles were also used to test the stability of multiple regression analysis results, and the linear tests were performed by assigning medians to each GPR tertile as a continuous variable in the models^20^.

An E-value was used to explore the potential of unmeasured confounding between GPR and 30-day mortality. The E-value was defined as the required magnitude for an unmeasured confounder to overturn the observed association between GPR and 30-day mortality^21^.

### Ethics approval and consent to participate

Ethics of the previous study was approved by the Norway regional committee (Regional Committee for Medical and Health Research Ethics South East) and informed consent was exempt for anonymous data. Thus, our secondary analysis based on this original study did not require separate ethical approval. And our study was carried out following all the relevant guidelines and regulations.

## Results

### Baseline characteristics of participants

The participants’ average age of participants was 77.64 ± 12.32 (range 34–100) years and 54.68% were female. The baseline characteristics and co-morbidities of participants are demonstrated in Table 1 by GPR tertiles. Serum-phosphate tertiles, atrial fibrillation/atrial flutter, cancer, pneumonia, and 30-day mortality of the GPR tertiles groups were statistically different (all P < 0.05). Large-artery atherosclerosis, cardioembolism, small-vessel occlusion, other determined etiology, and undetermined.

### Univariate analysis in relation to 30-day mortality

The 30-day mortality was chosen as a dependent variable, and univariate analysis were performed to determine which covariables were related to 30-day mortality. The results indicated that age (OR 1.10, 95% CI 1.057–1.13, P < 0.0001), department (Surgical *vs* Medical: OR 19.11, 95% CI 1.71–213.26, P = 0.0165), GPR (OR 2.18, 95% CI 1.31–3.63, P = 0.0028), serum-glucose (OR 1.33, 95% CI 1.14–1.55, P = 0.0003), serum-albumin (OR 0.82, 95% CI 0.78–0.87, P < 0.0001), hypertension (yes *vs* no: OR 0.50, 95% CI 0.26–0.96, P = 0.0378), heart failure (yes vs no: OR 5.44, 95% CI 2.22–13.28, P = 0.0002), chronic renal failure (yes *vs* no: OR 4.51, 95% CI 2.06–9.89, P = 0.0002), pneumonia (yes *vs* no: OR 9.17 , 95% CI 4.52–18.63, P < 0.0001) were associated with 30-day mortality (Table 2).

**Table 2:** Univariate analysis in relation to 30-day mortality.

Variables	Statistics	OR (95% CI)	P value
Age (year)	77.64 ± 12.32	1.10 (1.07, 1.13)	< 0.0001
**Gender**			
Male	341 (43.49%)	Reference	
Female	443 (56.51%)	1.28 (0.79, 2.08)	0.3242
**Department**			
Medical	781 (99.62%)	Reference	
Surgical	3 (0.38%)	19.11 (1.71, 213.26)	0.0165
**Ischemic stroke subtypes (TOAST)**			
Large-artery atherosclerosis	71 (9.06%)	Reference	
Cardioembolism	70 (8.93%)	0.49 (0.12, 2.02)	0.3204
Small-vessel occlusion	30 (3.83%)	1.67 (0.43, 6.39)	0.4564
Other determined etiology	2 (0.26%)	10.83 (0.60, 195.96)	0.1068
Undetermined	611 (77.93%)	1.22 (0.51, 2.94)	0.6520
GPR	1.59 ± 0.40	2.18 (1.31, 3.63)	0.0028
Serum-glucose (mmol/L)	6.46 ± 1.35	1.33 (1.14, 1.55)	0.0003
Serum-potassium (mmol/L)	4.12 ± 0.42	1.32 (0.76, 2.29)	0.3327
Serum-sodium (mmol/L)	139.50 ± 3.60	1.07 (0.99, 1.15)	0.0859
Serum-calcium (mmol/L)	2.35 ± 0.12	0.20 (0.03, 1.48)	0.1159
Serum-albumin (g/L)	39.21 ± 3.87	0.82 (0.78, 0.87)	< 0.0001
**Serum-phosphate tertiles (mmol/L), n (%)**			
Tertile1 (≤ 0.97)	85 (10.84%)	Reference	
Tertile2 (0.98–1.12)	88 (11.22%)	1.52 (0.59, 3.93)	0.3874
Tertile3 (≥ 1.13)	100 (12.76%)	1.44 (0.57, 3.65)	0.445
Not recorded	511 (65.18%)	0.88 (0.40, 1.95)	0.7611
**Serum-magnesium tertiles (mmol/L), n (%)**			
Tertile1 (≤ 0.78)	87 (11.10%)	Reference	
Tertile2 (0.79–0.84)	89 (11.35%)	0.97 (0.37, 2.59)	0.9594
Tertile3 (≥ 0.85)	107 (13.65%)	1.52 (0.64, 3.64)	0.3431
Not recorded	501 (63.90%)	0.79 (0.37, 1.69)	0.5492
**Hyperlipemia, n (%)**			
No	754 (96.17%)	Reference	
Yes	30 (3.83%)	–	^§^
**Hypertension, n (%)**			
No	593 (75.64%)	Reference	
Yes	191 (24.36%)	0.50 (0.26, 0.96)	0.0378
**AF, n (%)**			
No	575 (73.34%)	Reference	
Yes	209 (26.66%)	1.40 (0.84, 2.32)	0.1973
**Heart failure, n (%)**			
No	761 (97.07%)	Reference	
Yes	23 (2.93%)	5.44 (2.22, 13.28)	0.0002
**Chronic renal failure, n (%)**			
No	751 (95.79%)	Reference	
Yes	33 (4.21%)	4.51 (2.06, 9.89)	0.0002
**Acute renal failure**			
No	766 (97.70%)	Reference	
Yes	18 (2.30%)	1.90 (0.54, 6.71) 0.3197	0.3197
**COPD, n (%)**			
No	774 (98.72%)	Reference	
Yes	10 (1.28%)	2.36 (0.49, 11.34)	0.2819
**CHD, n (%)**			
No	742 (94.64%)	Reference	
Yes	42 (5.36%)	1.28 (0.49, 3.35)	0.6195
**Cancer, n (%)**			
No	770 (98.21%)	Reference	
Yes	14 (1.79%)	1.57 (0.34, 7.14)	0.5611
**Malnutrition, n (%)**			
No	766 (97.70%)	Reference	
Yes	18 (2.30%)	1.17 (0.26, 5.18)	0.8373
**Dehydration, n (%)**			
No	755 (96.30%)	Reference	
Yes	29 (3.70%)	1.52 (0.51, 4.48)	0.8598
**Pneumonia, n (%)**			
No	748 (95.41%)	Reference	
Yes	36 (4.59%)	9.17 (4.52, 18.63)	< 0.0001

Data were depicted as OR (95% CI) P-value. Serum-phosphate tertiles: Tertile1: ≤ 0.97 mmol/L; Tertile2: 0.98–1.12 mmol/L; Tertile3: ≥ 1.13 mmol. Serum-magnesium tertiles: Tertile1: ≤ 0.78 mmol/L; Tertile2: 0.79–0.84 mmol/L; Tertile3: ≥ 0.85 mmol/L.

*GPR* serum glucose to potassium ratio, *AF* atrial fibrillation/atrial flutter, *COPD* chronic obstructive pulmonary disease, *CHD* coronary heart disease.

^§^The model failed because of the small sample size.

### Multivariate logistic regression analysis of GPR and 30-day mortality

In multivariate regression analysis, we built three models adjusting for different covariates to verify the stability of the results. The results of the crude model without adjusting for confounder factors showed that GPR and 30-day mortality were positively correlated (OR 2.18, 95% CI 1.31–3.63).Model I which was adjusted for age and gender also indicated the same association (OR 2.09, 95% CI 1.24–3.54). Model II which was further adjusted for age, gender, department, serum sodium, serum albumin, serum-magnesium tertiles, hypertension, heart failure, chronic renal failure, and pneumonia also revealed that GPR was independently associated with 30-day mortality (OR 2.01, 95% CI 1.12–3.61) (Table 3).

**Table 3:** Multivariate regression analysis of GPR and 30-day mortality.

Exposure	Crude model	Model I	Model II
GPR (continuous)	2.18 (1.31, 3.63) 0.0028	2.09 (1.24, 3.54) 0.0058	2.01 (1.12, 3.61) 0.0190
**GPR tertiles**			
Tertile 1	Reference	Reference	Reference
Tertile 2	1.22 (0.64, 2.33) 0.5523	1.28 (0.66, 2.50) 0.4638	1.24 (0.60, 2.56) 0.5626
Tertile 3	2.11 (1.17, 3.83) 0.0135	2.17 (1.17, 4.01) 0.0138	2.15 (1.09, 4.24) 0.0271
P for trend	0.0075	0.0089	0.0188

Data were depicted as OR (95% CI) P-value.

GPR: Tertile 1: ≤ 1.372; Tertile 2: 1.375–1.658; Tertile 3: ≥ 1.659.

*GPR* glucose to potassium ratio.

Crude model: not adjusted.

Mode I: Adjusted for age and gender.

Mode II: Adjusted for age, gender, department, serum sodium, serum albumin, serum-magnesium tertiles, hypertension, heart failure, chronic renal failure, and pneumonia.

### Curve fitting and two-piecewise linear regression model of GPR and 30-day mortality

**Figure 2:**
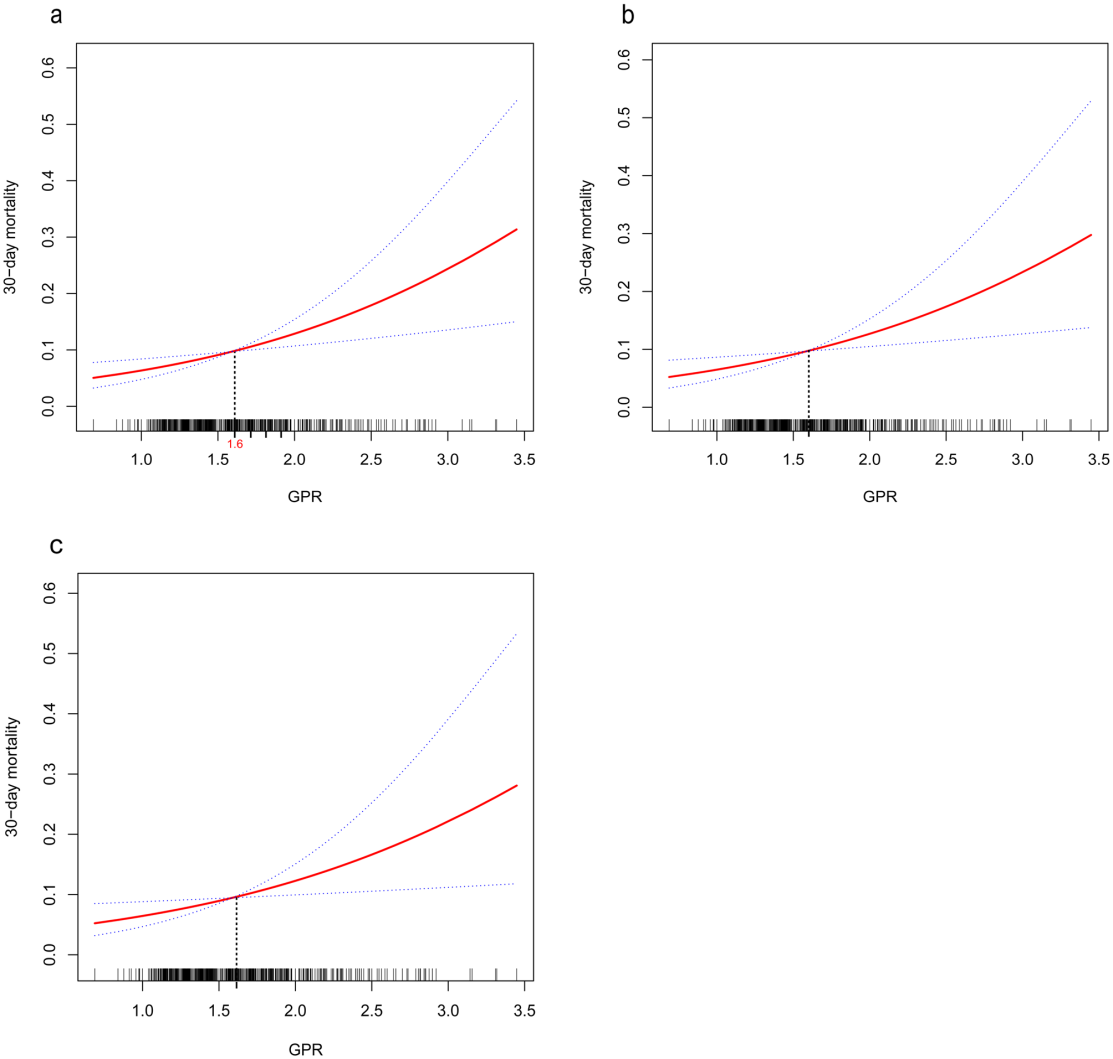
Multivariate adjusted smooth curve-fitting for association between GPR and 30-day mortality. *GPR* glucose to potassium ratio. (**a**) Crude model: not adjusted. (**b**) Mode I: adjusted for age and gender. (**c**) Mode II: adjusted for age, gender, department, serum sodium, serum albumin, serum-magnesium tertiles, hypertension, heart failure, chronic renal failure, and pneumonia. The red line represents the best-fit line, and the blue lines are 95% confidence intervals. The potential demarcation points are 1.6 according to the smoothing spline plots.

Curve fitting analysis, was adjusted according to three models (crude model, model I, model II) all indicated that a curve that that continues to rise, and the inflection point was approximately 1.6 (Fig. 2). According to the inflection point (GPR = 1.6), two-piecewise linear regression analysis models for different confounding factors (crude model, model I, model II) were used to explore the potential non-liner relationship. The result of model II showed two different effective sizes in two-piecewise linear regression equations, but the P-value for likelihood ratio test was 0.55 which was not statistically significant. The other two models showed similar results and the P-values for the likelihood ratio test were all > 0.05 (Table 4). Thus, the relationship of GPR with 30-day mortality was linear.

**Table 4:** Two-piecewise linear regression analysis for GPR and 30-day mortality.

GPR	Crude model	Model I	Model II
< 1.6	1.79 (0.35, 9.24) 0.4871	2.52 (0.46, 13.81) 0.2863	3.37 (0.56, 20.38) 0.1862
≥ 1.6	2.33 (1.12, 4.87) 0.0242	1.96 (0.90, 4.25) 0.0880	1.66 (0.70, 3.94) 0.2472
P-value for likelihood ratio test	0.805	0.820	0.550

Data were depicted as OR (95% CI) P-value.

*GPR* serum glucose to potassium ratio.

Crude model: not adjusted.

Mode I: Adjusted for age and gender.

Mode II: Adjusted for age, gender, department, serum sodium, serum albumin, serum-magnesium tertiles, hypertension, heart failure, chronic renal failure, and pneumonia.

### Sensitivity analysis

GPR (tertiles) were also plugged into the multiple regression equation for sensitivity analysis. The results of GPR as categorical variable (tertile) were consistent with the results of GPR as a continuous variable, the top tertile had 115% increment of diabetes risk when compared with the bottom tertile in the full model (model II), and found that the trend across the tertiles was significant (P for trend = 0.0188). Other two models (crude model, model I) showed similar results (Table 3).

An E-value was calculated to assess the sensitivity to unmeasured confounding. The primary findings were stable unless an unmeasured confounder existed and high positively related to GPR (OR ≥ 3.43) and 30 day-mortality (OR ≥ 3.43).

## Discussion

To the best of our knowledge, this is the first study to explore the relationship between GPR and the clinical IS outcome. Our study showed a significantly positive correlation between GPR levels and 30-day mortality. Further, the stability of the association was verified by adjusting for potential confounding factors (mode I, OR 2.09, 95% CI 1.24–3.54; model II, OR 2.01, 95% CI 1.24–3.54). In sensitivity analysis, we handled GPR as a categorical variable (tertiles) and the results showed an increasing trend of OR values from tertile 1 to tertile 3 in the three models (P values for trend all < 0.05). Moreover, the curve fittings of GPR levels and 30-day mortality showed a gradual upward curve in smoothing plots for the three different models (Supplementary Fig. 1). According to the inflection point in the curve fitting plot, two-piecewise linear regression analyses with three different adjustment methods were performed and all the results showed a linear relationship between GPR and 30-day mortality.

GPR is a novel parameter that can be measured quickly in clinics. Fujiki et al. first reported the potential association between baseline GPR and H–K grade and Glasgow score at discharge in a retrospective cohort study including 565 aSAH patients^10^. In another study, they investigated cerebral vasospasm after aSAH, and reported that elevated GPR levels were related to cerebral vasospasm grades and ischemic events induced by cerebral vasospasm^22^. In addition, the roles of GPR levels in other acute neurological injury related disease including acute intracerebral hemorrhage. Neuropsychiatric syndrome after carbon monoxide poisoning, and severe traumatic brain injury, have been proven^11–13^. In these studies, the baseline GPR levels were all observed in worse clinical outcome group than the normal group. Our results added evidence with regard to association between GPR levels and short-term outcome (30-day mortality) in cerebral ischemic injury. The results of the aforesaid studies suggested that GPR levels were closely related to pathological neurological disorders.

Hyperglycemia is very common in the acute phase of IS, even among non-diabetic IS patients^23^. The phenomenon of post-stroke hyperglycemia was believed to be a type of stress hyperglycemia induced by high cortisol and catecholamine levels after ischemic injury^24^. In addition, stress hyperglycemia had been suggested to be associated with stroke severity. Patients with stress hyperglycemia often had more serious strokes than those with type 2 diabetes mellitus (T2DM)^25^. Guo et.al have reported that IS patients with stress hyperglycemia had a higher risk of 90-day stroke recurrence than those with T2DM^26^. However, inconsistent results were shown in studies to explore the association between hyperglycemia and clinical outcomes of IS patients^25,27–29^. A study by Zonneveld et.al showed that stress hyperglycemia was associated with post-stroke infections and poor functional outcome^27^. Further, among both IS patients treated with intravenous thrombolysis^28^ and those treated via mechanical thrombectomy^29^, stress hyperglycemia was proven to be associated with a poor outcome. However, Tziomalos et al. believed that stress hyperglycemia was correlated with stroke severity rather than directly being related to an adverse outcome^25^. Besides, a recent clinical trial showed that glucose-lowering therapy did not help in improving the prognosis^30^. In addition to the heterogeneity of study design, and the potential non-linear relationship between admission serum glucose and outcome reported previously^31^, the complicated and multifaceted path mechanism underlying stress hyperglycemia may lead to the discordance of results. Likewise, as another important clinical blood biomarker, serum potassium levels play a crucial role in maintaining basic cellular functions. Normally, potassium ion is mostly stored in the cells and transported outside the membrane by sodium/potassium ATPase when necessary. Some population-based evidences have demonstrated that a potassium-rich diet could lower the risk of stroke^32,33^. Nevertheless, existing studies investigating the association between serum potassium and stroke outcome have shown contrasting results^34–36^. In our univariate analysis, admission serum potassium was not associated with 30-day mortality. This may be because of the intermediate factors, such as serum glucose, which impacted the relationship between serum potassium and short-term outcome. Accordingly, current research on the specific relationship between serum potassium level and the outcome of IS patients is still limited.

Despite these observations investigating the baseline GPR levels and IS outcome, the mechanisms underlying these findings still remain unknown. In severe stress injury conditions, sympathetic activation would result in an increased secretion of stress hormones including catecholamines, growth hormone, cortisol, and cytokines, and then induce a hyperglycemic response and insulin resistance^24^. In acute IS patients, the regulation of sodium/potassium ATPase by high catecholamine levels and secretion of insulin all would lead to potassium influx^37^. Thus, post-stroke hyperglycemia and hypokalemia may reflect the stress-related activation and a disorder of the hypothalamic–pituitary–adrenal (HPA) axis. HPA axis dysregulation believed to play a key role in the process of successive energy pump failure and various signaling cascades of IS^38^. In addition, a high cortisol level would activate the renin–angiotensin–aldosterone system (RAAS) to induce low serum potassium^39^. Brown et.al also thought that lower serum potassium level may represent increased activity of RAAS^40^. Current evidences show that RAAS plays a pivotal role in the progression of IS^41^, and angiotensin II receptors blockers could help to stroke prevention^42^. Based on the abovementioned discussion and considering the combined effects of serum potassium and serum glucose levels, the GPR index may be a good indicator for reflecting the status of HPA axis and RAAS dysregulation after IS. Moreover, studies of other stress damage types including acute myocardial infarction^43^, blunt abdominal trauma^44^, pulmonary embolism^45^, and even intermediate syndrome induced by anticholinesterase-containing chemicals poisoning^46^ all showed a stable correlation between increased GPR and poor outcomes or more severe symptoms. These studies also suggest that GPR may be a potential marker of stress injury for reflecting the condition of the whole body in severe disease.

The pathogenesis of IS is complex, and there was limited knowledge concerning it until now. Therefore, most treatment strategies currently followed that were developed by targeting known key pathogenetic links are often inadequately effective. The role of stress responses^38^ and stress-related markers^3^ has been proven to play a pivotal role in IS progression and has gained increasing attention. Our results suggested that comprehensive treatments including appropriate potassium supplementation, hypoglycemic treatment, and stress response blocker (β-blocker, RAAS inhibitor) may improve the short-term prognosis with high GPR levels. However, because of the nature of this retrospective study, the causality of high GPR levels and short-term outcome in IS patients could not be established. Future studies were needed to explore the causal relationship and verify the effectiveness of these treatments. In addition, because the raw data of our study were from a large emergency cohort, GPR may have a broad application prospect for IS patients admitted in emergency departments, especially in primary or smaller emergency departments as a brief blood biomarker, information on which could quickly be obtained at admission.

## Conclusion

Among IS patients, GPR is positively correlated with 30-day mortality, and the relationship between them is linear. Thus, GPR at admission may be a promising predictor of the short-term outcome of IS patients.

### Strengths and limitations

There are several advantages to our study. First, the results of univariate analysis, regression coefficient change, and previous literature were used to select covariates. Second, curve fitting and two-piecewise linear regression analysis were performed to explore the potential non-liner relationship, which had been shown in a previous study. Third, one crude model and three models which had been adjusted for potential confounding variables were used to test the stability of the results. Fourth, to avoid the contingency of analysis, GPR was considered as a continuous variable and categorical variable in the multiple regression equation, and sensitivity analysis and trend test were performed.

However, this study also has some limitations. First, the presence of unmeasured confounders could not be excluded. Since the secondary analysis originated from a retrospective cohort, variables that were not collected could not be adjusted. E-value was used to explore the potential for unmeasured confounding between GPR and 30-day mortality and the result showed that an unmeasured confounder was unlikely to explain the entirety of the mortality effect. Second, ICD-10 codes for renal failure may not be clear enough to identify renal function status, and hospitalization information after emergency admission including intensive care unit duration and length of hospital stay were not included in the analysis; future studies collecting renal function indicators (serum creatinine and baseline eGFR), intensive care unit duration, and length of hospital stay are required to more accurately explore the association and mechanisms. Second, there was no record concerning the levels of serum hormones such as catecholamines, glucagon, and corticosteroids in the original data; thus, we could not clarify the reason for high GPR in patients with severe IS. Third, lacking treatment information before the first blood tests (dextrose, potassium, or insulin) and after admission may lead to bias. However, given that the treatment would tend to a bias toward the null, we believed that the unmeasured confounding of medication treatment may underestimate the observed effect. Fourth, though first-time laboratory results at admission, which are more likely to reflect the initial state of the patient at the onset, were used, it would be better to examine the dynamic changes in GPR in future studies to understand the potential mechanism of the associations. Because of the retrospective study design, we could not confirm the time of blood collection, which will influence the GPR level. Thus, further prospective studies with predesigned identical examination time are required. Finally, the participants of this study are Norwegian populations, and the findings do not necessarily apply to other populations.

## Supplementary Information

Supplementary Information.

## Data Availability

The data are available from the ‘DataDryad’ database (www.datadryad.org).

## Abbreviations

GPR: Serum glucose to potassium ratio
IS: Ischemic stroke
aSAH: Aneurysmal subarachnoid hemorrhage
AF: Atrial fibrillation/atrial flutter
COPD: Chronic obstructive pulmonary disease
CHD: Coronary heart disease
