# Supplementary material for: The association between serum glucose to potassium ratio on admission and short-term mortality in ischemic stroke patients
Source: Sci Rep. 2022 May 17;12:8233. doi: 10.1038/s41598-022-12393-0 (PMC9114007; doi:10.1038/s41598-022-12393-0)
Supplement: Supplementary file 1 — Supplementary Information. [file 41598_2022_12393_MOESM1_ESM.docx]

Supplementary Material

**Supplementary Table 1**.Pooled estimates of multivariate regression analysis based on five multiple imputation data

| Exposure | Crude model | Mode Ⅰ | Mode Ⅱ |
| --- | --- | --- | --- |
| GPR | 2.18 (1.31, 3.63) 0.002723 | 2.09 (1.24, 3.53) 0.005875 | 2.00 (1.12, 3.56) 0.019509 |

GPR=serum glucose to potassium ratio

Crude model: not adjusted.

Mode Ⅰ: Adjusted for age and gender.

Mode Ⅱ: Adjusted for age, gender, department, serum sodium, serum albumin, serum-magnesium tertiles, hypertension, heart failure, chronic renal failure, and pneumonia.

**Supplementary Table 2.** The comparison of GPR and 30-day mortality between missing and no-missing serum-phosphate groups

| Serum-phosphate | Missing group | Non-missing group | P-value |
| --- | --- | --- | --- |
| N=784 | 511 | 273 |  |
| GPR | 1.58 ± 0.40 | 1.61 ± 0.40 | 0.268 |
| 30-day mortality |  |  | 0.380 |
| no | 458 (89.63%) | 250 (91.58%) |  |
| yes | 53 (10.37%) | 23 (8.42%) |  |

**Supplementary Table 3.** The comparison of GPR and 30-day mortality between missing and no-missing serum-phosphate groups

| Serum-magnesium | Missing group | Non-missing group | P-value |
| --- | --- | --- | --- |
| N=784 | 501 | 283 |  |
| GPR | 1.57 ± 0.39 | 1.62 ± 0.43 | 0.104 |
| Death within a month |  |  | 0.374 |
| no | 450(89.29%) | 258(91.17%) |  |
| yes | 51 (10.18%) | 25(8.83%) |  |

**Supplemental Figure 1.** **The process of multiple imputation**

**
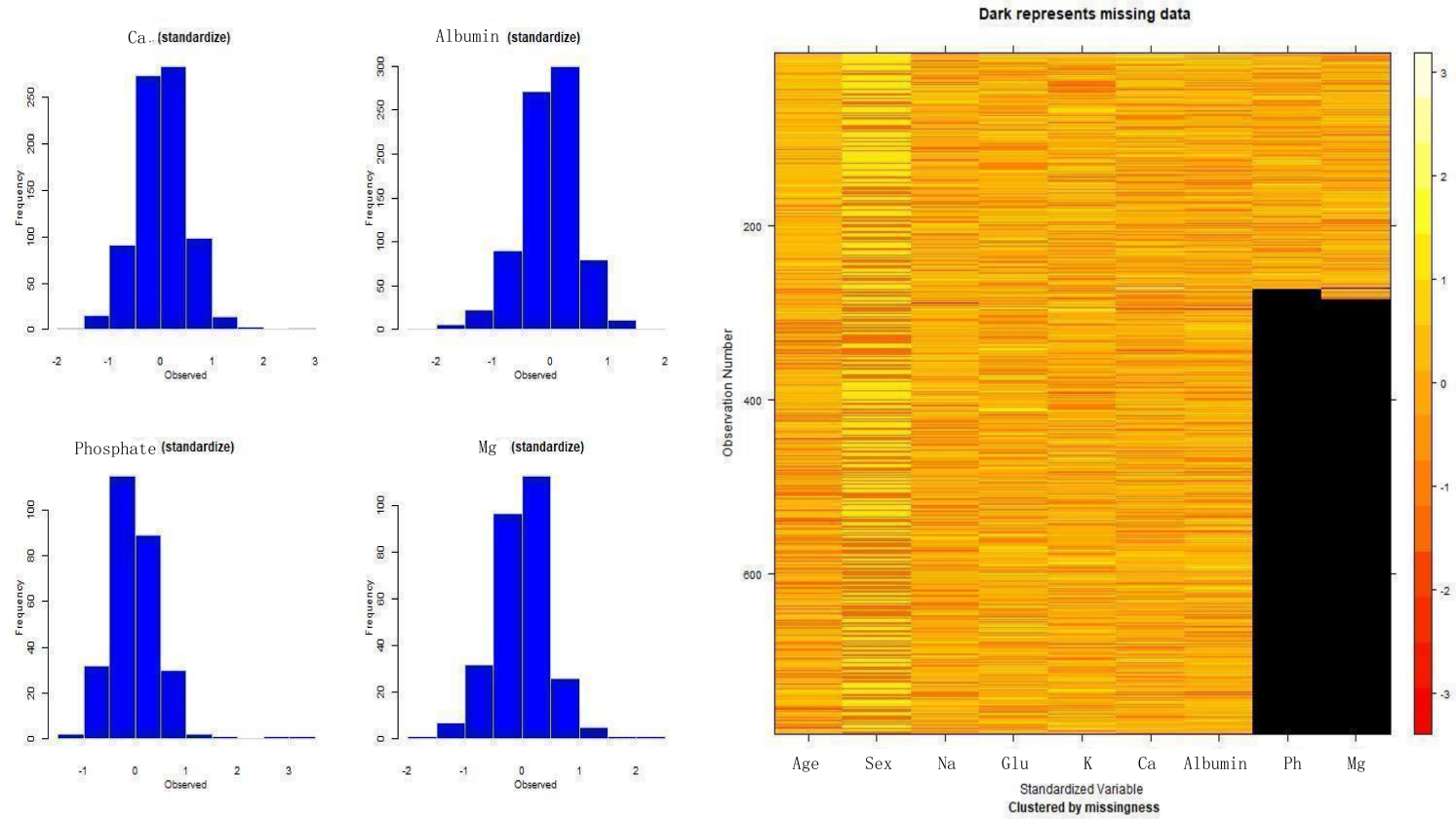
**
